# Peer Review of “Determinants of Periodic Health Examination Uptake: Insights From a Jordanian Cross-Sectional Study”

**DOI:** 10.2196/71529

**Published:** 2025-02-05

**Authors:** Ali Ahmed

**Affiliations:** 1 Division of Infectious Diseases and Global Public Health, School of Medicine, University of California, San Diego, La Jolla, CA, United States

**Keywords:** periodic health examination, PHE, preventive health services, routine health checkups, Jordan, cross-sectional study

*This is a peer-review report for “Determinants of Periodic Health Examination Uptake: Insights From a Jordanian Cross-Sectional Study.”*

## Round 1 Review

### Specific Comments

#### Major Comments

1. In this manuscript [1], write in detail about the data collection procedure.
2. Why was a convenience sampling technique employed?
3. “All collected data are treated with strict confidentiality.” Some language corrections are required.

#### Minor Comments

There are a lot of formatting issues; many things seem copied and pasted.
